# Declining Prevalence of Human Immunodeficiency Virus (HIV)–Associated Neurocognitive Disorders in Recent Years and Associated Factors in a Large Cohort of Antiretroviral Therapy–Treated Individuals With HIV

**DOI:** 10.1093/cid/ciac658

**Published:** 2022-08-19

**Authors:** Ilaria Mastrorosa, Carmela Pinnetti, Anna Clelia Brita, Annalisa Mondi, Patrizia Lorenzini, Giulia Del Duca, Alessandra Vergori, Valentina Mazzotta, Roberta Gagliardini, Marta Camici, Federico De Zottis, Marisa Fusto, Maria Maddalena Plazzi, Elisabetta Grilli, Rita Bellagamba, Stefania Cicalini, Andrea Antinori

**Affiliations:** Clinical Department of Infectious Diseases and Research, HIV/AIDS Unit, National Institute for Infectious Diseases Lazzaro Spallanzani Istituto di Ricovero e Cura a Carattere Scientifico, Rome, Italy; Clinical Department of Infectious Diseases and Research, HIV/AIDS Unit, National Institute for Infectious Diseases Lazzaro Spallanzani Istituto di Ricovero e Cura a Carattere Scientifico, Rome, Italy; Clinical Department of Infectious Diseases and Research, Psychology Service, National Institute for Infectious Diseases Lazzaro Spallanzani Istituto di Ricovero e Cura a Carattere Scientifico, Rome, Italy; Clinical Department of Infectious Diseases and Research, HIV/AIDS Unit, National Institute for Infectious Diseases Lazzaro Spallanzani Istituto di Ricovero e Cura a Carattere Scientifico, Rome, Italy; Clinical Department of Infectious Diseases and Research, HIV/AIDS Unit, National Institute for Infectious Diseases Lazzaro Spallanzani Istituto di Ricovero e Cura a Carattere Scientifico, Rome, Italy; National Center for Disease Prevention and Health Promotion, National Institute of Health, Rome, Italy; Clinical Department of Infectious Diseases and Research, HIV/AIDS Unit, National Institute for Infectious Diseases Lazzaro Spallanzani Istituto di Ricovero e Cura a Carattere Scientifico, Rome, Italy; Clinical Department of Infectious Diseases and Research, HIV/AIDS Unit, National Institute for Infectious Diseases Lazzaro Spallanzani Istituto di Ricovero e Cura a Carattere Scientifico, Rome, Italy; Clinical Department of Infectious Diseases and Research, HIV/AIDS Unit, National Institute for Infectious Diseases Lazzaro Spallanzani Istituto di Ricovero e Cura a Carattere Scientifico, Rome, Italy; Clinical Department of Infectious Diseases and Research, HIV/AIDS Unit, National Institute for Infectious Diseases Lazzaro Spallanzani Istituto di Ricovero e Cura a Carattere Scientifico, Rome, Italy; Clinical Department of Infectious Diseases and Research, HIV/AIDS Unit, National Institute for Infectious Diseases Lazzaro Spallanzani Istituto di Ricovero e Cura a Carattere Scientifico, Rome, Italy; Clinical Department of Infectious Diseases and Research, HIV/AIDS Unit, National Institute for Infectious Diseases Lazzaro Spallanzani Istituto di Ricovero e Cura a Carattere Scientifico, Rome, Italy; Clinical Department of Infectious Diseases and Research, HIV/AIDS Unit, National Institute for Infectious Diseases Lazzaro Spallanzani Istituto di Ricovero e Cura a Carattere Scientifico, Rome, Italy; Clinical Department of Infectious Diseases and Research, HIV/AIDS Unit, National Institute for Infectious Diseases Lazzaro Spallanzani Istituto di Ricovero e Cura a Carattere Scientifico, Rome, Italy; Clinical Department of Infectious Diseases and Research, HIV/AIDS Unit, National Institute for Infectious Diseases Lazzaro Spallanzani Istituto di Ricovero e Cura a Carattere Scientifico, Rome, Italy; Clinical Department of Infectious Diseases and Research, HIV/AIDS Unit, National Institute for Infectious Diseases Lazzaro Spallanzani Istituto di Ricovero e Cura a Carattere Scientifico, Rome, Italy; Clinical Department of Infectious Diseases and Research, HIV/AIDS Unit, National Institute for Infectious Diseases Lazzaro Spallanzani Istituto di Ricovero e Cura a Carattere Scientifico, Rome, Italy; Clinical Department of Infectious Diseases and Research, HIV/AIDS Unit, National Institute for Infectious Diseases Lazzaro Spallanzani Istituto di Ricovero e Cura a Carattere Scientifico, Rome, Italy

**Keywords:** AIDS, HIV-associated neurocognitive disorders (HAND), HIV, integrase strand transfer inhibitor, neurocognitive impairment

## Abstract

**Background:**

HIV-associated neurocognitive disorders (HAND) have been suggested as persistent even with effective antiretroviral therapy (ART). Aims were to evaluate HAND prevalence and associated factors, in a large cohort of people-with-HIV (PWH).

**Methods:**

ART-treated PWH, underwent a neuropsychological examination through a battery of 12 tests exploring 5 different domains, between 2009 and 2020, were included in this cross-sectional analysis. HAND were classified according to Frascati's criteria. Participants were defined as complaining or not-complaining if a cognitive complaint was reported or not. Chi-square for trend and multivariable logistic regression were fitted.

**Results:**

Overall, 1424 PWH were enrolled during four three-years periods. HAND prevalence was 24%; among complainers (572/1424), it was 38%, higher than among not-complainers (15%). Over the study period, a decreasing HAND prevalence was found in the entire population (*P* < 0.001) and in complaining (*P* < 0.001); in not-complaining it remained stable (*P* = 0.182). Factors associated with HAND were older age, lower educational level, lower current CD4^+^ T-cell count and HCV co-infection. Compared to nonnucleoside reverse transcriptase inhibitors, receiving dual and integrase strand transfer inhibitor (INSTI)-based therapies was associated with a decreased risk of HAND, as well as being tested in more recent years.

**Conclusions:**

In this large cohort of ART-treated PWH, mostly virologically suppressed, a remarkable decreasing HAND prevalence was observed. Besides HIV- and patient-related factors, the reduced risk of HAND found with dual and INSTI-based regimens along with a more recent ART initiation, could suggest a potential role of new treatment strategies in this decline, due to their greater virologic efficacy and better tolerability.

## Teaser

Over the last decade, a decreasing prevalence of human immunodeficiency virus (HIV)–associated neurocognitive disorders (HAND) was found. The asymptomatic form remained prevalent compared with symptomatic stages. A reduced risk for HAND was observed with dual and integrase strand transfer inhibitor–based regimens, along with more recent initiation of antiretroviral therapy.

The introduction of combination antiretroviral therapy (cART) has successfully changed the clinical features of people with human immunodeficiency virus (HIV; PWH), leading to dramatic improvements in HIV-related morbidity and mortality. Despite these benefits, neurocognitive impairment (NCI) has remained a relevant area of concern for PWH. Possible explanations are the persistent viral replication in the central nervous system (CNS) and the resultant immune activation, irreversible brain injury prior to initiation of cART, longer life expectancy and prolonged exposure to a greater risk of age-associated conditions (eg, metabolic or cardiovascular comorbidities), and possible ART neurotoxicity [1, 2]. A clear improvement in neurological outcomes in the era of cART has been achieved with a significant decline in the rate of HIV-associated dementia (HAD); however, high rates of mild to moderate NCI still exist [3–8]. Some longitudinal studies have shown that current HIV-associated neurocognitive disorders (HAND) are no longer progressive neurologic syndromes as they were in the pre-cART era [9, 10]. The prevalence of HAND needs to be elucidated because of its variable rates in different cohorts, depending on the target population and the methods of NCI assessment. Over the last decade, a variety of estimates have been reported, exceeding or close to 50% in certain studies from North America [6, 10] and lower in studies from Europe [11, 12]. Recently, a global prevalence of 43% was estimated [8]. When specific subgroups of patients or particular settings are considered, these percentages may vary, such as among certain transmission groups [9, 13], patients who are treated early [14], aviremic patients [15], or clinical trial participants [16]. In previous studies, several risk factors have been implicated with the incidence and evolution of NCI, as the level of immunosuppression, defined as nadir CD4^+^ T-cell count [6, 8, 17, 18], or HIV virologic control [6, 10, 19], orduration of HIV infection [14], or the specific ART regimen, concerning CNS penetration and ART toxicity [12, 20, 21]. In addition to these HIV-related factors, sociodemographic characteristics such as older age, race, and education level [7–12, 22–25] and comorbid conditions such as diabetes, cardiovascular disease, metabolic syndrome [7, 26, 27], and depression [5, 22] may influence the likelihood of developing HAND.

In this study, we included a participant sample broadly representative of patients being followed in a reference research Italian hospital, without strict inclusion criteria. The primary aim was to estimate HAND prevalence in more recent years of cART. The secondary aim was to identify potential factors associated with HAND among several sociodemographic, clinical, laboratory, and therapeutic variables.

## METHODS

### Study Design and Study Participants

Participants were recruited at the National Institute for Infectious Diseases Lazzaro Spallanzani in Rome, Italy. Between January 2009 and December 2020, all PWH receiving cART and referred for neuropsychological assessment (NPA) for clinical or research purposes were considered eligible for the study and were prospectively enrolled. Individuals with altered NPA who reported NCI in the presence of confounding conditions likely to contribute to the impairment were excluded. Here, we present the cross-sectional analysis of the first NPA performed for each participant over the study period. Reasons for the neuropsychological evaluation included suspected NCI by clinical history or clinical examination, possible increased risk of NCI due to associated factors (eg, low CD4^+^ T-cell count nadir, older age, HIV-related noninfectious comorbidities), and enrollment in research studies with NPA included per the protocol. Signs or symptoms of suspected NCI were assessed by a physician using a 3-question screening test, developed by Simioni et al [15] and listed as one possible assessment in the European AIDS Clinical Society guidelines [28]. Participants with a positive screening test, indicating a cognitive complaint, were defined as “complaining”; those with a negative screening test were defined as “not complaining.” All participants enrolled in research studies signed the specific informed consent, and protocols were approved by the institute’s ethics committee. All other patients were enrolled in the Pre-IMP Study, which evaluated factors associated with NCI in a cohort of PWH, taking or not cART, including both a retrospective and a prospective design.

### Study Procedures

For all study participants, epidemiological and demographic data, concomitant medications, and clinical data related to HIV disease and ART were collected and anonymously recorded. After clinical evaluation and laboratory tests, all participants were examined by 2 trained neuropsychologists (A. C. B. and G. D. D.). NPA was carried out through a standardized and comprehensive battery of 12 tests to explore 5 cognitive domains. HAND was defined according to the Frascati’s criteria, after excluding participants with confounding conditions likely to contribute to the impairment [3] and with a foreign native language and no command of the Italian language. Moreover, as a measure of the neurocognitive performance (NCP), *z* scores for each neuropsychological test and cognitive domain and a global a global neuropsychological z-score (NPZ-12) score were calculated. Among participants with HAND and NCI, the presence of a trend over time was also assessed. Details on NPA, HAND, and NCI definitions and *z* scores are provided in the Supplementary Materials.

### Data Analyses

Descriptive characteristics were provided using medians and interquartile ranges (IQRs) for continuous variables and frequencies and percentages for categorical variables. Complaining and not-complaining patients’ characteristics were compared using the Wilcoxon Mann–Whitney test for continuous variables and *χ*^2^ test for categorical variables. Additionally, the *χ*^2^ test for trend across ordered group was applied to assess for the presence of a trend over time, divided in 3-year periods. A logistic regression model, uni- and multivariable, was fitted using the presence of any stage of HAND as the outcome and all the main confounders as covariates. A statistically significant difference in the variables tested was indicated by a *P* value <.05 (2-sided). Statistical analysis was performed using STATA 15.1 software.

## RESULTS

### Population Characteristics

Among 1424 ART-treated PWH, 2383 neuropsychological tests were performed during the entire study period (2009–2020); for this analysis, we considered the first test for each individual. Overall, 572 of 1424 (40%) NPA were performed after a cognitive complaint in deficit of memory, attention, or concentration; for all other tests (852 of 1424, 60%), cognitive complaints were not reported by the participant or the physician. The main characteristics of the study population at NPA are described in Table 1. Briefly, they were mainly male (81%), nearly half were men who have sex with men (MSM; 44%), and 26% were aged >55 years. Participants had a good viroimmunological status (HIV-1 RNA <50 copies/mL in 80%, with a median CD4^+^ count of 575 cell/mm^3^ (interquartile range [IQR], 380–764), a long history with HIV (9 years; IQR, 3–19), and a high level of instruction (13 years; IQR, 8–15); anti-hepatitis C virus (HCV) antibodies were positive in 24%. At NPA, the majority of participants were receiving a triple therapy: 41% nonnucleoside reverse transcriptase inhibitor (NNRTI)–based, 21% boosted protease inhibitor (bPI)–based, and 18% integrase strand transfer inhibitor (INSTI)–based (16%). The remaining participants were on a bPI mono (5%) or dual (9%; 6% INSTI-based) therapy. The 2 groups of participants (complaining and not-complaining) were significantly different at baseline.

**Table 1.** General Characteristics of the Study Population at Neuropsychological Assessment and Comparison Between the 2 Groups of Complaining and Not-Complaining Patients

Characteristic	Overall (N = 1424)	Not-Complaining (n = 852, 59.8%)	Complaining (n = 572, 40.2%)	*P V*alue
Male gender, n (%)	1158 (81.3)	715 (83.9)	443 (77.5)	.002
Age, years,				
ȃMedian (IQR)	49 (41–55)	50 (41–55)	48 (42–55)	.934
ȃ<45, n (%)	507 (35.6)	309 (36.3)	198 (34.6)	.777
ȃ45–55, n (%)	545 (38.3)	325 (38.2)	220 (38.5)	
ȃ>55, n (%)	372 (26.1)	218 (25.6)	154 (26.9)	
Mode of HIV transmission, n (%)				
*ȃ*Men who have sex with men	631 (44.3)	400 (47.0)	231 (40.4)	<.001
ȃHeterosexuals	301 (21.1)	188 (22.1)	113 (19.8)	
ȃIntravenous drug users	388 (27.2)	197 (23.1)	191 (33.4)	
ȃOther/unknown	104 (7.3)	67 (7.9)	37 (6.5)	
Years of education, median (IQR)	13 (8–15)	13 (10–16)	13 (8–13)	.003
Years with HIV				
ȃMedian (IQR)	8.9 (2.5–19.0)	9.47 (3.1–19.3)	7.8 (1.8–18.3)	.115
ȃ<5, n (%)	506 (35.5)	286 (33.6)	220 (38.5)	.113
ȃ5–10, n (%)	250 (17.6)	158 (18.5)	92 (16.1)	
ȃ>10, n (%)	630 (44.2)	389 (45.7)	241 (42.1)	
ȃMissing, n (%)	38 (2.7)	19 (2.2)	19 (3.3)	
Hepatitis C virus serostatus, n (%)				
ȃNegative	918 (64.5)	523 (61.4)	395 (69.1)	.005
ȃPositive	337 (23.7)	226 (26.5)	111 (19.4)	
ȃUnknown	169 (11.9)	103 (12.1)	66 (11.5)	
Nadir CD4^+^ cells/mm^3^, n (%)				
ȃMedian (IQR)	248 (119–378)	276 (166–394)	199 (82–339)	<.001
ȃ<200, n (%)	511 (35.9)	224 (26.3)	287 (50.2)	<.001
ȃ≥200, n (%)	812 (57.0)	534 (62.7)	278 (48.6)	
ȃMissing, n (%)	101 (7.1)	94 (11.0)	7 (1.2)	
CD4^+^ cells/mm^3^ at NPA				
ȃMedian (IQR)	575 (383–764)	605 (433–790)	512 (297–726)	<.001
ȃ≤350, n (%)	309 (21.7)	135 (15.9)	174 (30.4)	<.001
ȃ351–500, n (%)	249 (17.5)	151 (17.7)	98 (17.1)	
ȃ501–700, n (%)	402 (28.2)	270 (31.7)	132 (23.1)	
ȃ>700, n (%)	444 (31.2)	290 (34.0)	154 (26.9)	
ȃMissing, n (%)	20 (1.4)	6 (0.7)	14 (2.4)	
HIV-RNA copies/mL at NPA, n (%)				
ȃ>50	264 (18.5)	130 (15.3)	134 (23.4)	<.001
ȃ ≤50	1135 (79.7)	713 (83.7)	422 (73.7)	
ȃMissing	25 (1.8)	9 (1.1)	16 (2.8)	
Type of current regimen, n (%)				
ȃNRTI + NNRTI	586 (41.2)	411 (48.2)	175 (30.6)	<.001
ȃNRTI + bPI	301 (21.1)	132 (15.5)	169 (29.6)	
ȃNRTI + INSTI	223 (15.7)	143 (16.8)	80 (14.0)	
*ȃ*Raltegravir	83 (3.5)	28 (3.5)	55 (3.4)	.915
*ȃ*Elvitegravir	78 (3.3)	28 (3.5)	50 (3.1)	.606
*ȃ*Dolutegravir	123 (5.2)	40 (5.1)	83 (5.2)	.871
*ȃ*Bictegravir	145 (6.1)	10 (1.3)	135 (8.5)	<.001
*ȃ*Dual therapy	125 (8.8)	60 (7.0)	65 (11.4)	<.001
*ȃ*Raltegravir	45 (1.9)	24 (3.0)	22 (1.4)	.006
*ȃ*Dolutegravir	62 (2.6)	28 (3.5)	34 (2.1)	043
*ȃ*No INSTI-based	81 (3.4)	42 (5.3)	38 (2.4)	<.001
ȃbPI monotherapy	66 (4.6)	44 (5.2)	22 (3.8)	<.001
ȃOther	123 (8.6)	62 (7.3)	61 (10.7)	<.001
Years of NPA				
ȃ2009–2011	244 (17.1%)	110 (12.9%)	134 (23.4%)	<.001
ȃ2012–2014	414 (29.1%)	230 (27.0%)	187 (32.2%)	
ȃ2015–2017	292 (20.5%)	167 (19.6%)	125 (21.8%)	
ȃ2018–2020	474 (33.3%)	345 (40.5%)	129 (22.6%)	

*P* values at Wilcoxon Mann–Whitney test or *χ*^2^ test, as appropriate, are shown. Abbreviations: bPI, boosted protease inhibitor; HIV, human immunodeficiency virus; INSTI, integrase strand transfer inhibitor; IQR, interquartile range; NNRTI, nonnucleoside reverse transcriptase inhibitor; NPA, neuropsychological assessment; NRTI, nucleoside reverse transcriptase inhibitor.

### HAND Prevalence

**Figure 1.**
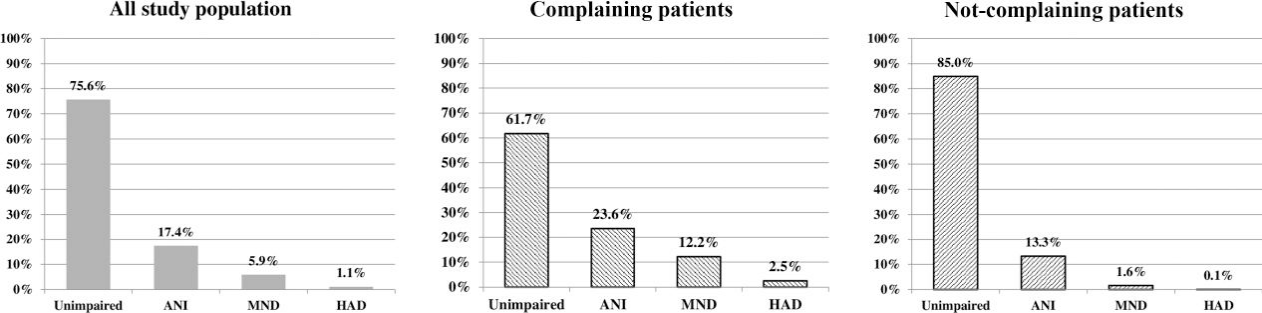
Human immunodeficiency virus–associated neurocognitive disorder prevalence for the entire study population, complaining and not-complaining patients. Abbreviations: ANI, asymptomatic neurocognitive impairment; HAD, human immunodeficiency virus–associated dementia; MND, mild neurocognitive disorder.

Overall, HAND prevalence was 347 of 1424 (24%), and each HAND stage was distributed as follows: asymptomatic neurocognitive impairment (ANI), 248 (17%); mild neurocognitive disorder (MND), 84 (6%); and HAD, 15 (1%). The remaining 1077 (76%) patients were classified as neurocognitively unimpaired. We found a different prevalence of HAND in the complaining and not-complaining groups: 219 of 572 (38%) and 128 of 852 (15%), respectively (Figure 1).

**Figure 2.**
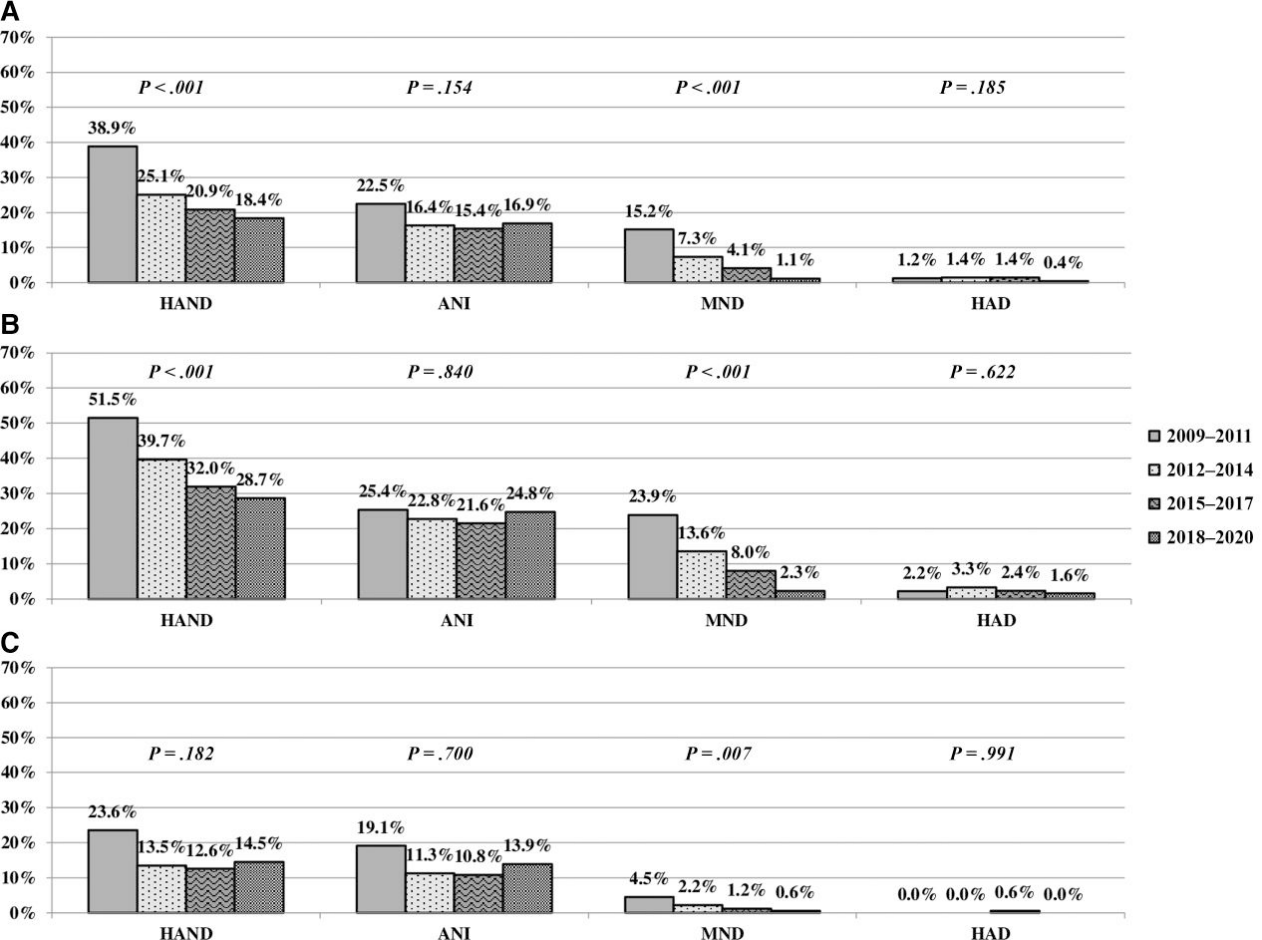
HAND prevalence according to calendar period of neuropsychological assessment for the entire study population *(A)*, complaining patients *(B)*, and not-complaining patients *(C)*. *P* values at *χ*^2^ for trend are shown. Abbreviations: ANI, asymptomatic neurocognitive impairment; HAD, human immunodeficiency virus–associated dementia; HAND, human immunodeficiency virus–associated neurocognitive disorder; MND, mild neurocognitive disorder.

**Figure 3.**
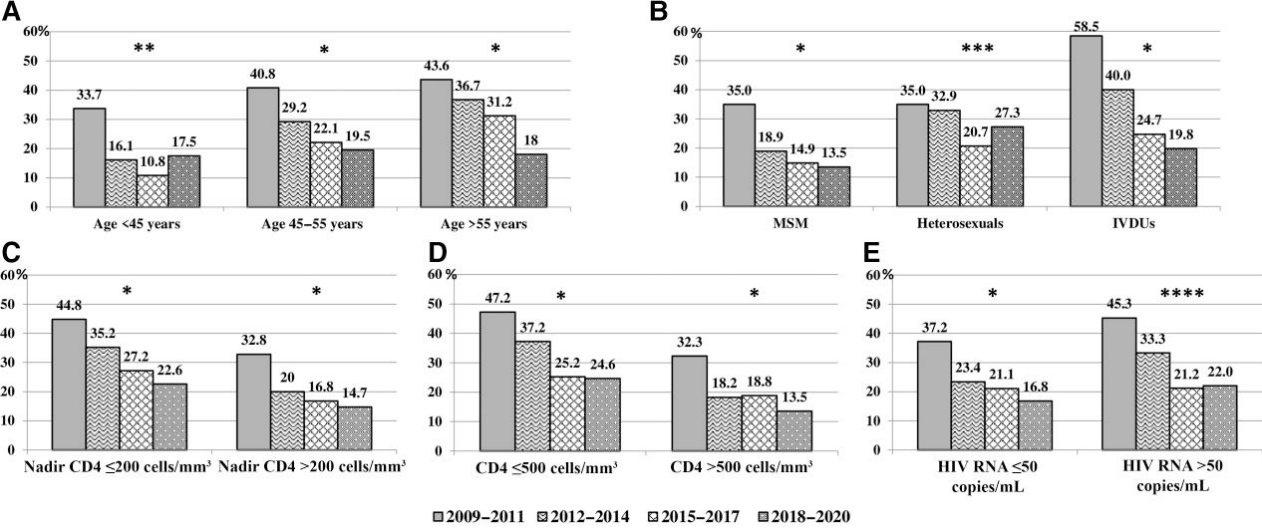
Human immunodeficiency virus–associated neurocognitive disorder prevalence for the entire study population according to calendar period of neuropsychological assessment (NPA), after stratifying for age *(A)*, mode of HIV transmission *(B)*, nadir *(C)* and current CD4^+^ T-cell count *(D)*, and HIV-RNA at NPA *(E)*. *P* values at Chi square for trend are shown. *P value <.001 at *χ*^2^ for trend. ** = .013, *** = .068, **** = .002. Abbreviations: HIV, human immunodeficiency virus; IVDU, intravenous drug user; MSM, men who have sex with men.

Considering the entire population of 1424 participants in all 4 time periods (2009–2011, 2012–2014, 2015–2017, 2018–2020), HAND prevalence decreased over time, from 39% to 18% (*P* < .001). A similar trend was observed for MND frequencies (*P* < .001); while ANI and HAD showed a stable prevalence (*P* = .154 and *P* = .185, respectively; Figure 2A). When the 2 groups of participants were analyzed, we observed the same decreasing trend for complaining patients (from 52% to 29%, *P* < .001); whereas, among not-complaining patients, HAND prevalence changed from 24% to 15% (*P* = .182). Regarding HAND severity, only MND showed a decreasing prevalence over time in both groups of complainers and not-complainers (*P* < .001 and *P* = .007, respectively). ANI remained the most common diagnosis with a stable frequency (*P* = .840 for complaining and *P* = .700 for not-complaining patients), and the low prevalence of HAD was confirmed across the study period (*P* = .622 for complaining and *P* = .991 for not-complaining patients; Figure 2B, 2C). The declining trend in HAND prevalence was confirmed after stratifying for age, mode of HIV transmission (with the exception of the heterosexual’s group, which showed only a trend toward a decrease in HAND prevalence over time; *P* = .068), nadir and current CD4^+^ T-cell count, and HIV-RNA at NPA (Figure 3).

### Neurocognitive Performance

The NCP was lower among complaining patients compared with not-complaining patients (Supplementary Table 1). During the study period, we did not find any statistical evidence for a change in the global NCP (Supplementary Table 2). See the Supplementary Materials for detailed results.

### Factors Associated With HAND

Demographic characteristics, such as older age and a lower educational level, were strongly associated with a higher risk of HAND diagnosis (adjusted odds ratio [AOR], 1.01; 95% confidence interval [CI)], 1.00–1.03; *P* = .030 and AOR, 0.84; 95% CI, .80–.86; *P* < .001, respectively), as well as having anti-HCV antibody positivity (AOR, 1.37; 95% CI, 1.02–1.985; *P* = .036). On the contrary, a progressively lower risk of having HAND was observed as the current CD4^+^ count increased (351–500 cells/mm^3^: AOR, 0.66; 95% CI, .46–.95; *P* = .027; 501–700 cells/mm^3^: AOR, 0.49; 95% CI, .34–.69; *P* < .001; and >700 cells/mm^3^: AOR, 0.43; 95% CI, .30–.62; *P* < .001). Regarding ART regimens prescribed at NPA, participants receiving a dual regimen and an INSTI-based triple therapy were associated with a decreased risk of having HAND compared with those on an NNRTI-based triple therapy (AOR, 0.54; 95% CI, .34–.87; *P* = .010 and AOR, 0.68; 95% CI, .47–.99; *P* = .046, respectively). Finally, to be tested in more recent years significantly predicted a reduced risk of HAND diagnosis (2015–2017: AOR, 0.49; 95% CI, .34–.72; *P* < .001 and 2018–2020: AOR, 0.41; 95% CI, .28–.61; *P* < .001). No evidence was found for a significant association with the other confounders investigated (gender, mode of HIV transmission, years from HIV diagnosis, nadir CD4^+^ count and HIV-RNA at NPA; Table 2).

**Table 2.** Factors Associated With Human Immunodeficiency Virus–Associated Neurocognitive Disorders by Uni- and Multivariable Logistic Regression

	Odds Ratio	95% Confidence Interval		*P* Value	Adjusted Odds Ratio	95% Confidence Interval		*P* Value
Gender, Female vs Male	1.11	.82	1.50	.508				
Age, 10-year increase	1.29	1.14	1.45	<.001	1.01	1.00	1.03	.**030**
Mode of HIV transmission								
ȃHeterosexuals	1.00	…		…	1.00	…	…	…
ȃMen who have sex with men	0.55	.41	.74	<.001	1.19	.91	1.57	.206
ȃIntravenous drug users	0.92	.66	1.29	.635	0.88	.63	1.22	.451
*ȃ*Other/unknown	0.78	.48	1.28	.328	1.28	.79	2.07	.318
Education, 1-year increase	0.83	.80	.86	<.001	0.84	.81	.86	**<**.**001**
Years of HIV infection								
ȃ<5	1.00	…		…	1.00	…	…	…
ȃ5–10	1.25	.87	1.80	.226	1.27	.90	1.79	.179
ȃ>10	1.44	1.09	1.91	.011	1.23	.91	1.67	.187
ȃMissing	3.21	1.63	6.30	<.001	3.45	1.73	6.87	<.001
Hepatitis C virus serostatus								
ȃNegative	1.00	…		…	1.00	…	…	…
ȃPositive	1.47	1.11	1.94	.008	1.37	1.02	1.85	.**036**
ȃUnknown	1.28	.88	1.86	.200	1.29	.85	1.96	.238
Nadir CD4^+^, cell/mm^3^								
ȃ≥200	1.00	…		…	1.00	…	…	…
ȃ<20	1.98	1.53	2.55	<.001	1.05	.81	1.37	.720
ȃMissing	1.14	.69	1.89	.601	1.25	.71	2.22	.441
CD4^+^ cell/mm^3^ at NPA								
ȃ≤350	1.00	…		…	1.00	…	…	…
ȃ351–500	0.55	.38	.79	.001	0.66	.46	.95	.**027**
ȃ501–700	0.43	.31	.60	<.001	0.49	.34	.69	**<**.**001**
ȃ>700	0.34	.25	.48	<.001	0.43	.30	.62	**<**.**001**
ȃMissing	0.40	.13	1.24	.113	0.42	.08	2.29	.318
HIV-RNA copies/mL at NPA								
ȃ≤50	1.00	…		…	1.00	…	…	…
ȃ>50	1.39	1.03	1.87	.030	1.04	.73	1.48	.835
ȃMissing	1.05	.41	2.65	.923	1.40	.31	6.31	.665
Type of current regimen								
ȃNRTI + NNRTI	1.00	…		…	1.00	…	…	…
ȃNRTI + bPI	2.23	1.63	3.04	<.001	1.16	.86	1.57	.344
ȃNRTI + INSTI	0.99	.68	1.46	.968	0.68	.47	.99	.**046**
ȃDual therapy	1.08	.67	1.73	745	0.54	.34	.87	.**010**
ȃbPI monotherapy	0.96	.51	1.82	.907	0.72	.43	1.20	.205
ȃOther	1.50	.96	2.34	.074	1.17	.74	1.85	.501
Years of NPA								
ȃ2009–2011	1.00	…		…	1.00	…	…	…
ȃ2012–2014	0.53	.37	.74	<.001	0.74	.53	1.04	.084
ȃ2015–2017	0.41	.28	.61	<.001	0.49	.34	.72	**<**.**001**
ȃ2018–2020	0.35	.25	.50	<.001	0.41	.28	.61	**<**.**001**
Cognitive complaint	3.51	2.73	4.52	<.001	3.81	3.00	4.85	**<**.**001**

The multivariable model was fitted retaining variables from the univariable exploration if they had a *P* value <.05. *P* values <.05 are shown in bold in the adjusted analysis.

Abbreviations: bPI, boosted protease inhibitor; HIV, human immunodeficiency virus; INSTI, integrase strand transfer inhibitor; NPA, neuropsychological assessment; NNRTI, nonnucleoside reverse transcriptase inhibitor; NRTI, nucleoside reverse transcriptase inhibitor.

## DISCUSSION

We assessed neurocognitive function using a comprehensive cognitive testing battery in 1424 ART-treated PWH, mostly virologically suppressed, over a period of 12 years. We found an overall HAND prevalence of 24%, declining from 39% to 18% in more recent years. This decreasing trend was confirmed among complaining patients, whereas HAND prevalence remained stable in individuals with no specific cognitive complaints.

Our results on HAND prevalence in the study population are in contrast with the higher estimates recently reported worldwide [8] and previously found in both the CNS HIV Antiretroviral Therapy Effects Research (CHARTER) cohort [6, 10] and a small study conducted among PWH with long-standing undetectable HIV-RNA [15]. Our findings are in line with the estimates described in Europe [11, 12], in certain groups such as MSM [9, 13], and early diagnosed and managed PWH [14]. Additionally, despite important differences in the NPA performed and NCI definition, a similar low prevalence was described in 2 studies conducted among ART-treated PWH with both well-controlled HIV infection [29] and not [25]. Finally, in the early cART era, a higher estimate of NCI (238 of 432, 55%) was observed in a previous study from our hospital [18]. In the present study, it is likely that the characteristics of the enrolled population and the ART regimens prescribed contributed to these different estimates. Indeed, all participants were receiving ART, including more recent regimens, and they were mainly men, about half MSM and more than half HCV-seronegative, with a relatively high level of instruction and with an optimal viroimmunological control, all factors known to be associated with a better NCP. Consistent with previous evidence in the cART era that showed a low rate of HAD but still high rates of milder forms of HAND [3–8], as in our study, the diagnoses of ANI and MND were more frequent compared with the stable, low prevalence of HAD; for MND, a clear decreasing trend was found in all the 3 groups. Finally, when the change in HAND prevalence was analyzed by stratifying the population according to demographic and viroimmunological characteristics, the declining prevalence was further demonstrated. Despite the significant improvement in cognitive function of PWH in more recent years, the persistence of a substantial and stable rate of ANI was observed. This finding could partially support both the much debated high false-positive rate of the Frascati’s criteria and the questionable significance of the diagnosis of ANI, considering the unclear clinical impact of a premorbid condition and the lack of progression over the years [2, 30–32]. However, in our study, we provided an exhaustive neuropsychological evaluation, including 2 or more tests for each cognitive domain, analyzed by making a comparison with a control population of PWH and through a strict assessment for the presence of potential confounding comorbid conditions. Furthermore, certain studies have suggested that participants with an ANI diagnosis are more likely to progress to a symptomatic status [7, 33]. In addition, the early recognition of cognitive disorders made it possible to establish interventions that might slow the progression of the disease as ART introduction or optimization, control of some comorbidities (eg, cardiovascular diseases, diabetes), lifestyle modifications, or a rehabilitation strategy.

Interestingly, global NCP, as measured by NPZ-12, remained stable over time in participants with both HAND and NCI not defined by Frascati's criteria. Because NPZ-12 is influenced by the sum of the scores obtained in each test, having very high scores in some tests can compensate for lower scores without considering that cognitive functions in one area may already be damaged. In addition, NPZ-12 alone does not allow the assessment of a person's “functioning.” This finding sheds further light on the need to find a gold standard for defining NCI in PWH.

In our analysis, several factors were found to be associated with HAND development, most in line with previous observations. As expected, we identified some demographic characteristics of participants that may affect neurocognition. Within our population, age was associated with an increased risk of NCI, similar to previous studies [9, 11, 12, 22]. The prolonged exposure to HIV and to cART, together with age-related comorbidities and concomitant medication use, may interact and contribute to the development of NCI in older PWH [34]. A recent matched study involving aging individuals with and without HIV demonstrated the association between NCI and HIV in such a population [24]. Moreover, in our analysis, having a lower education level contributed to a worse NCP, confirming robust evidence in the literature showing the protective role of instruction [10–12, 23].

Interestingly, we demonstrated a reduced risk of having HAND in the case of the absence of anti-HCV antibodies. The available literature presents controversial evidence about whether HCV coinfection results in additive deleterious impacts on neurocognition in PWH [35–38] due to aspects such as HCV entry in the CNS, the consequent persistent inflammatory and immune activation status, and HCV-mediated liver dysfunction. We did not attempt to give a definitive answer based on our findings (eg, we did not consider the role of HCV active replication), but the findings suggest that patients without a history of HCV coinfection may be less affected by neurocognitive alterations.

With regard to immunological status, we observed a strong correlation between a higher CD4^+^ T-cell count at NPA and a better NCP without any association with CD4^+^ T-cell nadir. These results confirm the importance of cART initiation to achieve and maintain an optimal immunological recovery in order to reduce the prevalence and severity of HAND and to prevent any decline [8, 25, 33]. Although prior studies [6, 17, 18, 25] and a recent meta-analysis [8] have suggested that patients with low CD4^+^ T-cell count nadir have a high risk of developing NCI, we did not find evidence for this association, and the association is likely due to the features of our population who were enrolled in more recent years when fewer patients experienced a severe immunosuppression in their history.

In contrast with previous reports [6, 10, 19], in our adjusted analysis, we did not find any evidence for an association between virologic control and NCP, and 2 main reasons may be hypothesized. First, the high proportion of PWH with well-controlled viremia (80% in the entire population) may have mitigated the effect of this covariate in the adjusted model. Second, the potential persistence of HIV replication in the CNS, which was not evaluated in this study, despite suppression of plasma HIV-RNA, may be related to ongoing cognitive impairment [1, 2, 39]. We can consider these findings to be directly linked to the lower risk for HAND observed in this study, with certain antiretroviral therapies compared with NNRTI-based triple therapies. In particular, INSTIs are highly effective in both plasma and the CNS, resulting in suppression of HIV replication and, potentially, in better NCP. Moreover, when neuropsychiatric symptoms are reported with the use of INSTIs in several post-marketing studies, their effects, beneficial or detrimental, on NCP are not well clarified [12, 40–42]. In our analysis, INSTI-based regimens, prescribed in 20% of the study population (15% as triple and 5% as dual therapy), seemed to have a protective role in neurocognition in terms of reduced risk of HAND. Similarly, in our study, dual regimens, INSTI- or not INSTI-based (prescribed in 5% and 3%, respectively), were found to halve the risk of having HAND. According to our data, we cannot determine whether this result is directly related to a beneficial effect of the drugs used or, more likely, to the characteristics of the patients selected for dual therapies. Indeed, these therapies are generally prescribed in participants with optimal viroimmunological control, which has a well-described protective role for neurocognition. Finally, the safety profile and the high tolerability of both regimens, INST-based and dual, ensure optimal levels of ART adherence and enhance their effectiveness.

Despite the large number of tests considered and the great representativeness of the sample, together with the systematic NPA and the strict criteria applied to classify HAND, which are the main strengths of our analysis, our findings should be interpreted in the context of its limitations. Temporality and causation between factors of interest and the development of NCI are difficult to assess in a retrospective, observational study with a cross-sectional design; moreover, the absence of a well-matched HIV-negative control group did not allow us to compare prevalence and severity of NCI. For this analysis, we did not consider certain confounders that may affect neurocognition, such as comorbidities, years of untreated/uncontrolled HIV, previous ART regimens, reasons for therapy switch, drug adherence, or social aspects. Nevertheless, our findings are consistent with those from prior studies, suggesting that the observed results may be significant and clinically meaningful.

In conclusion, a remarkable decreasing prevalence of HAND was observed over the last decade in the entire population, and the mildest stage of HAND remained prevalent compared with symptomatic forms. In the absence of an appropriate screening method, a comprehensive NPA is recommended for patients with a cognitive complaint and may be suggested for those without in order to have the chance to provide targeted interventions. No individual agent or group of antiretrovirals has unequivocally showed benefits for preventing HAND. However, the consistent use of ART and the choice of new treatment strategies, dual or INSTI-based regimens, to guarantee optimal virologic control and avoid serious immunosuppression may have beneficial long-term effects in improving neurocognitive outcomes.

## Supplementary Data

Supplementary materials are available at Clinical Infectious Diseases online. Consisting of data provided by the authors to benefit the reader, the posted materials are not copyedited and are the sole responsibility of the authors, so questions or comments should be addressed to the corresponding author.

## Supplementary Material

ciac658_Supplementary_Data

## Notes

***Acknowledgments.*** The authors gratefully acknowledge the nurse staff, all study participants, and the doctors who took care of the patients.

***Financial support*.** This work was supported by Ministero della Salute (Italian Ministry of Health), Ricerca Corrente–Linea 2, sottoprogetto 3.
