# Supplementary material for: Declining Prevalence of Human Immunodeficiency Virus (HIV)–Associated Neurocognitive Disorders in Recent Years and Associated Factors in a Large Cohort of Antiretroviral Therapy–Treated Individuals With HIV
Source: Clin Infect Dis. 2022 Aug 19;76(3):e629–37. doi: 10.1093/cid/ciac658 (PMC9907497; doi:10.1093/cid/ciac658)
Supplement: ciac658_Supplementary_Data [file ciac658_supplementary_data.docx]

**SUPPLEMENTARY MATERIALS**

**Declining prevalence of HIV-associated neurocognitive disorders in more recent years and associated factors, in a large cohort of ART-treated HIV-infected individuals.**

Ilaria Mastrorosa^*^, Carmela Pinnetti, Anna Clelia Brita, Annalisa Mondi, Patrizia Lorenzini, Giulia Del Duca, Alessandra Vergori, Valentina Mazzotta, Roberta Gagliardini, Marta Camici, Federico De Zottis, Marisa Fusto, Maria Maddalena Plazzi, Elisabetta Grilli, Rita Bellagamba, Stefania Cicalini, Andrea Antinori.

**^*^Corresponding author:**

Ilaria Mastrorosa, MD

Clinical Department of Infectious Diseases and Research, HIV/AIDS Unit

National Institute for Infectious Diseases Lazzaro Spallanzani IRCCS

Via Portuense 292, 00149 Roma, Italy

E-mail: [ilaria.mastrorosa@inmi.it](mailto:ilaria.mastrorosa@inmi.it)

Phone: +39 06 55170546

Fax: +39 06 55170477

**Contents of Supplementary Materials**

**Supplementary methods, page 3**

1. Neuropsychological assessment, page 3
2. HIV-associated neurocognitive disorders (HAND) classification, page 4
3. Classification of comorbid conditions, page 5
4. Neurocognitive Performance, page 6

**Supplementary results, page 7**

1. Neurocognitive Performance, page 7

**References, page 9**

**Supplementary methods**

**I. Neuropsychological assessment**

Neuropsychological assessment was carried out through a standardized and comprehensive battery of 12 tests on 5 different cognitive domains:

1. speed of mental processing (Trail Making Test A, WAIS-R Digit Symbol);
2. mental flexibility (Trail Making Test B, Stroop test color-word, Phonemic verbal fluency-FAS);
3. working memory/short-term memory/concentration (WAIS-R Digit Span forward and backward, Corsi's Block-Tapping Test);
4. memory (RAVLT - Rey Auditory Verbal Learning Test, immediate and delayed recall);
5. fine motor functioning (Grooved Pegboard Test, dominant/non-dominant hand).

The assessment was conducted by two trained neuropsychologists (ACB, GDD). Raw scores derived from testing were converted to a demographically adjusted standard score (z-score). Participants were classified as neuropsychologically impaired or unimpaired based on their performance compared with normative data [2-7].

**II. HIV-associated neurocognitive disorders (HAND) classification**

As reported by the updated research nosology for HAND [1], patients were diagnosed as having asymptomatic neurocognitive impairment (ANI) or mild neurocognitive disorder (MND), if cognitive deficits involved two or more cognitive domains, over at least five analysed domains, not readily attributable to comorbid conditions, and documented by performances of at least one standard deviation below the mean for norms, without interference in everyday functioning if ANI, or with at least mild interference in daily functioning if MND. Finally, HAD was defined by cognitive deficits involving at least two cognitive domains with a performance at least two standard deviations below the mean for norm on neuropsychological test and with a marked interference in everyday functioning.

**III. Classification of comorbid conditions**

HAND diagnosis required that the neurocognitive impairment (NCI) and the functional disability were attributable directly to the effects of HIV rather than to pre-existing or co-occurring conditions. Participants with confounding conditions likely to contribute to the NCI were excluded from the diagnosis of HAND according to Frascati’s criteria [1]. These comorbidities included:

1. any history of brain opportunistic infections;
2. non-HIV-associated neurologic disease (systemic disease or co-infection with HCV);
3. history of major psychiatric disorder;
4. current alcohol or substance dependence;
5. remote traumatic brain injury.

Together with detailed information about the comorbid conditions themselves, clinical judgment about their severity and the timing of the cognition impairment or of the everyday functioning decreasing, were required. The neuropsychologists (BAC and DDG) performed the NPA and used these guidelines with all the available historical and clinical data, collected by the own physician of each patient and verified through medical charts, in order to diagnose HAND or to exclude it because of the presence of confounding conditions.

**IV. Neurocognitive Performance**

As a measure of the neurocognitive performance, z-scores for each neuropsychological test and each cognitive domain, and a global NPZ-12 score, summarizing the z-scores of all the 12 tests, were calculated for the entire population, and after distinguishing in *complaining* and *not-complaining* patients. Comparisons between these two group were made using t-Student test. Negative values are shown if the performance was below the mean.

Among participants with HAND, diagnosed according to Frascati’s criteria, and among those classified as having a Neurocognitive Impairment (NCI), the presence of a trend over time, divided in three-years periods, of the global neurocognitive performance (NPZ-12) was assessed by Chi-square test for trend across ordered group. Individuals were defined as having NCI, if they scored >1 standard deviation (SD) below the normal mean in at least 2 tests, or >2 SD below in 1 test.

**Supplementary results**

**I. Neurocognitive Performance**

*Complaining* patients obtained lower scores in each test of the neuropsychological battery when compared to the *not-complaining* group. We found less statistical evidence for a difference for two tests (Corsi's Block-Tapping Test and Rey Auditory Verbal immediate and delayed recall; *p*=0.639 and 0.075, respectively). Evaluating the cognitive performance in each cognitive domain and the global NPZ-12, the difference between the two groups resulted more evident (Table S1).

**Table S1. Neurocognitive performance measured by means of z-scores for each test and each cognitive domain, and by a global NPZ-12 score, assessed in the entire population and in the two groups of *complaining* and *not-complaining* patients. Negative values are shown if the performance was below the mean; *p*-values at t-Student test are shown.**

|  | **Not-complaining**  **(n=852)** | **Complaining**  **(n=572)** | **p-value** | **Overall**  **(n=1,424)** |
| --- | --- | --- | --- | --- |
| **Neuropsychological tests, mean (SD)** |  |  |  |  |
| Trail Making Test A | -0.004 (0.97) | -0.320 (0.88) | <0.001 | -0.130 (0.94) |
| WAIS-R Digit Symbol | -0.395 (0.90) | -0.737 (0.88) | <0.001 | -0.528 (0.91) |
| Trail Making Test B | 0.156 (0.46) | -0.036 (0.65) | <0.001 | 0.080 (0.55) |
| Phonemic verbal fluency | 1.022 (1.30) | 0.710 (1.39) | <0.001 | 0.899 (1.35) |
| Stroop test color-word | -0.160 (1.15) | -0.538 (1.46) | <0.001 | -0.310 (1.30) |
| WAIS-R Digit Span forward | 0.074 (0.97) | -0.173 (0.88) | <0.001 | -0.024 (0.94) |
| WAIS-R Digit Span backward | -0.048 (0.90) | -0.147 (0.91) | 0.044 | -0.088 (0.91) |
| Corsi's Block-Tapping Test | -0.312 (0.94) | -0.337 (0.92) | 0.639 | -0.323 (0.94) |
| Rey Auditory Verbal Learning Test | 0.489 (1.06) | 0.354 (1.15) | 0.025 | 0.435 (1.10) |
| Rey Auditory Verbal immediate and delayed recall | 0.378 (1.08) | 0.270 (1.17) | 0.075 | 0.335 (1.12) |
| Grooved Pegboard Test, dominant hand | 0.046 (1.39) | -0.619 (2.42) | <0.001 | -0.217 (1.90) |
| Grooved Pegboard Test, non-dominant hand | 0.050 (1.23) | -0.421 (2.28) | <0.001 | -0.136 (1.74) |
| **Cognitive domains, mean (SD)** |  |  |  |  |
| Speed of mental processing | -0.192 (0.76) | -0.498 (0.73) | <0.001 | -0.312 (0.76) |
| Mental flexibility | 0.309 (0.84) | -0.023 (1.24) | <0.001 | 0.176 (1.03) |
| Working memory/short-term memory/concentration | -0.083 (0.73) | -0.219 (0.73) | <0.001 | -0.138 (0.73) |
| Memory | 0.434 (1.02) | 0.312 (1.10) | 0.034 | 0.385 (1.05) |
| Fine motor functioning | 0.048 (1.21) | -0.496 (2.11) | <0.001 | -0.166 (1.65) |
| **Global Cognitive Performance, mean (SD)** |  |  |  |  |
| NPZ-12 | 0.133 (0.55) | -0.099 (0.75) | <0.001 | 0.041 (0.64) |
| **Abbreviations**: n, number; SD, standard deviation. | | | | |

In our population, 672/1424 (47%) individuals were classified as having NCI. During the study period, we did not find any change in the global neurocognitive performance, measured as NPZ-12, among participants with both NCI and HAND (*p*=0.178 and 0.974, respectively) (Table S2).

**Table S2. Global neurocognitive performance, as measured by NPZ-12 score, according to calendar period of neuropsychological assessment of the participants classified as having HIV-associated neurocognitive disorders, defined by Frascati’s criteria, and Neurocognitive Impairment; *p*-values at Chi square for trend are shown.**

|  | **Overall** | **2009/2011** | **2012/2014** | **2015/2017** | **2018/2020** | **p-value** |
| --- | --- | --- | --- | --- | --- | --- |
| **Global Cognitive Performance**  **(NPZ-12), mean (SD)** |  |  |  |  |  |  |
| NCI (n=672) | -0.41 (0.61) | -0.53 (0.63) | -0.41 (0.55) | -0.35 (0.50) | -0.40 (0.68) | 0,178 |
| HAND (n=347) | -0.91 (0.72) | -0.98 (0.76) | -0.91 (0.61) | -0.77 (0.52) | -0.95 (0.82) | 0.974 |
| **Abbreviations**: n, number; SD, standard deviation; NCI, Neurocognitive Impairment; HAND, HIV-associated neurocognitive disorders. | | | | | | |

**References**

1. Antinori A, Arendt G, Becker JT, et al. Updated research nosology for HIV-associated neurocognitive disorders. Neurology, **2007**; 69(18):1789–1799.
2. Carlesimo GA, Caltagirone C, Gainotti G, et al. Batteria per la valutazione del Deterioramento Mentale (parte II): standardizzazione e affidabilità diagnostica nell’identificazione di pazienti affetti da sindrome demenziale. Archivio di psicologia, neurologia e psichiatria, **1995**; 56(4):471-488.
3. Amodio P, Campagna F, Olianas S, et al. Detection of minimal hepatic encephalopathy: normalization and optimization of the Psychometric Hepatic Encephalopathy Score. A neuropsychological and quantified EEG study. J Hepatol, **2008**; 49:346-353.
4. Orsini A, Laicardi C. WAIS-R. Contributo Alla Taratura Italiana. Firenze: Giunti O.S. Organizzazioni Speciali, **1997**.
5. Monaco M, Costa A, Caltagirone C, Carlesimo G. Forward and backward span for verbal and visuo-spatial data: standardization and normative data from an Italian adult population. Neurological sciences: official journal of the Italian Neurological Society and of the Italian Society of Clinical Neurophysiology. Neurol Sci, **2013**; 34(5):749-54.
6. Trites RL. Neuropsychological Test Manual. Ottawa, Ontario, Canada: Royal Ottawa Hospital, **1977**.
7. Valgimigli S, Padovani R, Budriesi C, Leone ME, Lugli D, Nichelli P. Test di Stroop: dati normativi italiani di una versione cartacea per l'uso clinico. Giornale Italiano di Psicologia, **2010**; 37(4):945-953.
